# FEDI-CODE: A federated and causally-informed framework for dementia risk prediction using multi-site patient data

**DOI:** 10.1371/journal.pone.0351957

**Published:** 2026-06-24

**Authors:** Mohammad Moniruzzaman, Md Shahab Uddin, Ahsan Ahmed, Md Aktarujjaman, Mumtahina Ahmed, Ashifur Rahman

**Affiliations:** 1 Department of Computer Science, Maharishi International University, Fairfield, Iowa, United States of America; 2 Computing, Business and Engineering, Georgia Institute of Technology, Atlanta, Georgia, United States of America; 3 Information Technology and Management, Webster University, Webster Groves, Missouri, United States of America; 4 Department of CSE, Bangladesh University of Business and Technology, Dhaka, Bangladesh; Hallym University, KOREA, REPUBLIC OF

## Abstract

Early detection of dementia is critical for timely intervention and disease management, yet it remains a challenging task due to the fragmented nature of healthcare data and the need for privacy-preserving solutions. This paper proposes FEDI-CODE, a Federated and Causally Informed Dementia Estimation framework that integrates deep learning, federated learning, and counterfactual inference to predict dementia risk across distributed patient data sources. FEDI-CODE is designed to operate without centralizing sensitive medical data, enabling collaborative training across institutions while preserving privacy. It combines temporal modeling of longitudinal imaging and clinical data with individualized treatment effect estimation for modifiable risk factors such as alcohol consumption, weight, and cardiovascular indicators. A fusion module aggregates representations from each site to form a global prediction head. Extensive experiments on simulated multi-site dementia datasets demonstrate that FEDI-CODE achieves an accuracy of 83.7%, a precision of 83%, a recall of 81%, an F1-score of 82%, and an AUC-ROC of 0.86, outperforming standard federated models and deep learning baselines by notable margins. The model also generalizes well to external datasets, achieving 79.2% accuracy and 0.80 AUC-ROC, confirming its robustness. Furthermore, FEDI-CODE produces interpretable causal insights by estimating individual treatment effects, offering actionable clinical value. These results highlight FEDI-CODE as a scalable, interpretable, and privacy-aware solution for early dementia screening and personalized risk assessment.

## 1. Introduction

Dementia is a progressive neurological syndrome characterized by a decline in cognitive function that interferes with daily life and independence. Early diagnosis is critical for effective intervention, care planning, and improving patient outcomes. However, detecting dementia at an early stage remains a challenge due to its subtle onset, variability across individuals, and the fragmented nature of healthcare data. Traditional diagnostic methods often rely on centralized data collection, which may not be feasible in settings where data privacy regulations or institutional boundaries prevent data sharing. The WHO estimates that 5.4% of men over 65 and 8.1% of women over 65 have dementia, out of a total estimated 55 million persons globally. By 2030, this figure is predicted to rise to 78 million; by 2050, it will reach 139 million [1]. Between 60 and 80 percent of dementia cases are caused by Alzheimer’s disease (AD), making it the most prevalent cause of dementia.

In recent years, deep learning has demonstrated strong capabilities in medical diagnosis tasks, including disease classification and prognosis prediction. Concurrently, federated learning (FL) has emerged as a promising paradigm for training machine learning models across decentralized data sources without moving raw data. Despite its success in preserving privacy, most FL approaches in healthcare are limited to predictive tasks and do not account for causal relationships or intervention effects, which are crucial in clinical decision-making. Additionally, existing models often fail to integrate heterogeneous data modalities—such as time-series imaging data and structured health metrics—into a unified predictive framework. Sykes et al. [2] qualitatively examined the implementation and efficacy of the UK National Audit of Dementia across six hospitals using interviews, observations, and document analysis. Despite organizational commitment, the audit’s feedback loop often failed to engage frontline clinicians effectively. The study identifies a gap in translating national feedback into actionable local strategies, highlighting a need for theory-informed improvements in content delivery and organizational response. Vaghari et al. [3] introduce the BioFIND dataset, a large multi-site magnetoencephalography (MEG) resting-state dataset of 324 participants (MCI patients and controls) for early dementia research. MEG data—combined with MRI and standardized using the BIDS format—allow researchers to investigate early functional brain changes. The study benchmarks basic classification using machine learning but emphasizes the need for improved biomarker discovery using complex modeling and broader community involvement. London et al. [4] used routinely collected NHS patient records from two trusts (25,326 patients total) to develop predictive models (notably logistic regression, AUROC of 0.78) for identifying dementia patients most at risk of needing psychiatric inpatient or enhanced community care. The results demonstrated reliable early identification of high-risk patients, although further work is needed to refine predictors and expand model generalizability. Across these works, common gaps include the need for better integration of data into clinical workflows, improved interpretability and dissemination of feedback, and large-scale validation of predictive tools across diverse settings.

To address these gaps, we propose FEDI-CODE, a Federated and Causally Informed Dementia Estimation framework that integrates deep learning, federated learning, and counterfactual inference. The model is designed to operate across multiple institutional datasets without centralizing patient information. It captures temporal patterns from longitudinal imaging and clinical assessments, estimates individual treatment effects (ITEs) for modifiable risk factors, and fuses distributed representations through a global prediction head. This enables not only accurate dementia classification but also interpretable and actionable risk profiling. In this study, dementia risk is defined as the model-estimated probability of developing or being diagnosed with dementia given an individual’s multimodal health profile, denoted as P(Dementia=1∣X). Our work makes the following contributions:

- We introduce a novel architecture that combines LSTM-based temporal modeling with causal inference in a federated learning setup, tailored for dementia risk prediction.
- We implement a counterfactual inference module that estimates the potential impact of modifiable clinical factors on dementia risk at the individual level.
- We design a multi-layer fusion mechanism to integrate site-specific representations for global prediction while maintaining privacy and interpretability.
- We conduct extensive experiments on simulated multi-site data, demonstrating that FEDI-CODE outperforms existing baselines in accuracy, robustness, and causal insight.

The remainder of this paper is structured as follows. Section 2 reviews related work on dementia prediction, federated learning in healthcare, and causal inference models. Section 3 describes the proposed FEDI-CODE framework, including data preprocessing, federated training setup, local model architectures, and counterfactual inference mechanisms. Section 4 details the experimental setup and presents the results, including classification performance, causal effect estimation, ablation studies, and generalization analysis. Section 5 discusses the implications, limitations, and future directions based on the experimental findings. Finally, Section 6 concludes the paper by summarizing the contributions and outlining potential extensions of this work.

## 2. Related work

Deep learning methods have been widely applied to dementia detection tasks, particularly in processing structural MRI and cognitive assessment data. Convolutional Neural Networks (CNNs) have been used to extract imaging biomarkers for classifying Alzheimer’s disease and other neurodegenerative conditions. Recurrent architectures such as Long Short-Term Memory (LSTM) networks [5] have shown success in modeling longitudinal cognitive decline. Despite their predictive power, most existing models require centralized access to datasets, which limits their applicability in privacy-sensitive clinical settings. Furthermore, these models typically focus on outcome prediction alone and do not address the influence of modifiable risk factors. Several studies have explored federated learning (FL) for Alzheimer’s and dementia prediction. Pan et al. [6] used EHR data from 44,899 patients across six simulated sites to predict the progression from mild cognitive impairment (MCI) to Alzheimer’s using a personalized FL approach with LSTM models. Their method improved AUC by 6% compared to local models and identified key features like BMI and vitamin B12 but did not explore causal relationships or multimodal data integration. Sahid et al. [7] applied FL on 6400 T1-weighted MRI images using CNN models, achieving 84.75% accuracy and showing that increasing local training improves results while reducing communication. However, their method struggled with performance under extreme data imbalance and did not use real-world institutional data.

Lakhan et al. [8] introduced FDCNN-AS, which combines multiple data types (MRI, PET, blood, EEG, and questionnaires) and trains deep CNNs across distributed clinical nodes with security layers. They achieved 99% accuracy but primarily focused on age groups and did not include causal inference or interpretability in their framework. Recent advancements in dementia prediction have leveraged machine learning and deep learning techniques to improve early diagnosis. Basheer et al. [9] proposed a modified capsule network (MCapNet) using the OASIS dataset (373 × 15 features) to distinguish demented from non-demented individuals, achieving 92.39% accuracy. Their model emphasizes feature selection and hierarchical analysis but is limited to single-site data without considering causal relationships. Wang et al. [10] developed a deep learning model using LSTM-based recurrent neural networks on a longitudinal EHR dataset (n = 26,921) from Partners HealthCare to predict 6-month, 1-year, and 2-year mortality risks in dementia patients, achieving AUCs of 0.978, 0.956, and 0.943, respectively. Though effective for identifying patients needing palliative care, their model focuses on mortality rather than disease onset and lacks federated learning considerations. Chen et al. [11] proposed a deep learning model, DS-GAN, to synthesize diffusion tensor imaging (DTI)-based scalar maps—mean diffusivity (MD) and fractional anisotropy (FA)—from T1 MRI sequences to predict dementia in cerebral small vessel disease (SVD). Trained on 4998 subjects from the UK Biobank and validated across four independent SVD cohorts (SCANS, RUN DMC, PRESERVE, and NETWORKS), the synthetic maps demonstrated high structural similarity (SSIM > 0.89 for MD) and achieved comparable dementia prediction accuracy to ground-truth DTI maps (c-index 0.82), surpassing traditional white matter hyperintensity metrics.

Meanwhile, Ortiz-Perez et al. [12] developed a multimodal architecture integrating textual and audio data from the DementiaBank Pitt Corpus using CNNs and Transformers. Their system achieved 90.36% accuracy using only text and outperformed unimodal approaches when modalities were combined, highlighting the effectiveness of speech and language features for early detection. Lastly, Kim and Lim [13] applied a DNN model enhanced with PCA preprocessing to Korean national health records (KNHANES; N = 7031, age > 65), using behavioral and healthcare usage data, and achieved 85.5% AUC, demonstrating that non-clinical, large-scale behavioral data can support early dementia prescreening. However, all studies are limited in handling federated or causally informed data harmonization across institutions, lacking mechanisms to disentangle site-specific biases or causal pathways, which FEDI-CODE aims to address.

Federated learning (FL) has emerged as a promising framework for collaborative model training across multiple institutions while preserving data privacy. Several works have applied FL to medical imaging, electronic health records, and disease prediction tasks. In the context of dementia, FL has been explored to train models across geographically distributed hospitals without pooling data. However, these approaches typically adopt standard supervised learning objectives and do not incorporate causal reasoning or interpretability mechanisms. Additionally, most FL implementations assume homogeneous data formats and overlook temporal dependencies present in patient trajectories. Recent advancements in federated learning (FL) for healthcare underline the urgent need for privacy-preserving, personalized, and explainable AI models across decentralized data environments. Ducange et al. [14] explored Fed-XAI techniques for Parkinson’s disease progression using a real-world voice dataset (UPDRS scores), comparing interpretable TSK-FRBS models and post-hoc explainable MLP-NNs. While the neural networks achieved higher accuracy, fuzzy rule-based systems offered superior interpretability and transparency, especially under non-iid settings, highlighting the trade-off between performance and explainability and the absence of causal modeling in current FL-XAI approaches.

The FedCure framework by Sachin et al. [15] tackled heterogeneity in IoMT-based healthcare through personalized FL embedded in a cloud-edge architecture. Evaluated on use cases like retinopathy and maternal health, FedCure achieved high accuracy and low communication overhead by applying model personalization at the device level yet lacked temporal or causal inference integration, which is crucial for chronic disease progression modeling. Complementarily, Tripathy et al. [16] introduced FedHealthFog, a fog-enabled FL system addressing energy and latency constraints in IoT healthcare by deploying fog nodes as dynamic aggregators. Tested across multiple benchmarks, the system significantly reduced latency (up to 87%) and energy use (up to 58%) but did not address patient-specific personalization or causal feature learning. Collectively, these studies validate FL’s applicability in privacy-sensitive healthcare settings and expose gaps in causal interpretability, dementia-specific applications, and robust personalization across multi-site data sources—gaps that FEDI-CODE aims to bridge.

Causal inference aims to estimate the effects of interventions or treatments on patient outcomes and is critical for actionable healthcare recommendations. Techniques such as the Potential Outcomes Framework and Individual Treatment Effect (ITE) estimation have been applied to simulate clinical decisions and forecast outcomes under counterfactual scenarios. Models like TARNet [17] and DragonNet use deep learning to estimate counterfactuals, but they are typically trained on centralized datasets and do not operate under federated constraints. Moreover, these models are rarely integrated into disease-specific diagnostic pipelines, limiting their practical adoption in clinical environments. The studies have explored various causal and federated modeling approaches for dementia risk prediction but leave room for integrative, multi-site frameworks like FEDI-CODE.

Recent efforts have explored integrating causal inference into federated frameworks to enable both privacy preservation and individualized decision support. However, most of these approaches are still theoretical or limited to synthetic datasets. There is limited work combining causal modeling with multi-modal, real-world clinical data, particularly for complex neurodegenerative conditions like dementia. The lack of publicly implemented and evaluated systems that jointly model temporal health data, enable treatment effect estimation, and preserve data locality highlights a critical research gap. Mandal [18] introduces a vertical federated learning (VFL) framework tailored for Alzheimer’s detection, addressing HIPAA constraints by enabling multimodal training (e.g., demographic + MRI) across institutions without sharing raw data. Using OASIS and ADNI datasets, the model showed robust performance while preserving privacy, but it lacked causal inference and generalizability across more heterogeneous, real-world hospital systems. Kuo et al. [19] propose D-CLEF, a decentralized framework supporting both horizontally- and vertically-partitioned data using blockchain and distributed file systems. Applied to COVID-19, surgical, and myocardial datasets (n > 15,000 from UC Health and UK), D-CLEF matched centralized learning accuracy while outperforming siloed models, although synchronization time remained a trade-off; however, causal interpretability and dementia-focused applications were missing. Han et al. [20] present a federated causal inference method using summary statistics to estimate treatment effects across 51 U.S. hospitals (Medicare data, n = 11,103 AMI patients), showing up to 91% precision gains over local-only estimates and altering treatment conclusions in 63% of sites. Despite privacy and efficiency, the method did not focus on neurodegenerative diseases or multimodal data. Collectively, these studies highlight the need for a federated, causally grounded, privacy-preserving framework tailored to dementia risk prediction across diverse, multi-site datasets, a gap FEDI-CODE aims to fill.

In contrast to prior work, our proposed FEDI-CODE framework unifies federated learning, longitudinal deep modeling, and causal inference in a single architecture. It addresses both the technical challenges of decentralized training and the clinical need for interpretable, personalized risk assessment. By estimating how specific changes in patient health metrics could influence dementia progression, FEDI-CODE moves beyond prediction to support actionable intervention design in privacy-sensitive, multi-institution healthcare settings.

## 3. Methodology

**Fig 1:**
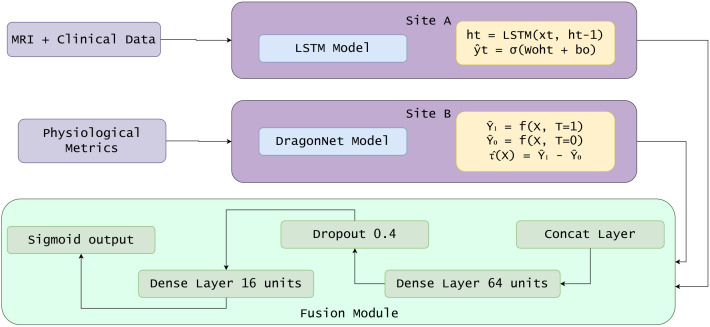
Our proposed FEDI-CODE: a federated and causally informed framework.

This section describes the proposed FEDI-CODE framework Fig 1, which integrates federated learning and counterfactual causal inference to model dementia risk using distributed patient datasets. The system is designed to simulate two distinct medical data silos: one containing imaging-derived and clinical data (Site A), and another containing physiological and behavioral health metrics (Site B). The model respects privacy constraints by enabling decentralized learning while incorporating causal estimation to evaluate modifiable risk factors.

### 3.1 Data preprocessing

To prepare the datasets for training the FEDI-CODE framework, we employed a structured preprocessing pipeline incorporating recent advances in deep learning-based data preparation. This process was applied independently to each data silo (Site A and Site B) to simulate the federated environment.

#### 3.1.1 Site A: MRI-derived and cognitive clinical data.

The dataset from Site A contains features such as eTIV, nWBV, CDR, MMSE, Age, and others extracted from longitudinal MRI sessions. We performed the following preprocessing steps:

**Normalization and outlier removal:** All continuous features were standardized using z-score normalization:


$$\begin{array}{c} z_{i}=\frac{x_{i}-\mu_{x}}{\sigma_{x}} \end{array}$$ (1)

Where, $x_{i}$ is the raw value, $\mu_{x}$ is the mean, and $\sigma_{x}$ is the standard deviation of the feature across all samples. This ensures that features are centered and scaled for stable convergence during training.

To handle outliers, we clipped values beyond three standard deviations:


$$\begin{array}{c} x_{i}^{\prime }=\min\left(\max(z_{i},-3),3\right) \end{array}$$ (2)

**Missing value imputation:** Some features such as SES contained missing values. We applied learned imputation using a denoising autoencoder:


$$\begin{array}{c} \hat{x}=D(E(x_{\text{masked}})) \end{array}$$ (3)

$$\begin{array}{c} {ℒ}_{\text{rec}}=\|x-\hat{x}{\|}^{2} \end{array}$$ (4)

where *E* and *D* are the encoder and decoder networks, respectively, trained to minimize reconstruction loss ${ℒ}_{\text{rec}}$ on partially masked inputs.

**Sequence construction:** To capture temporal changes in cognitive scores and brain volume, patient records were organized into sequences:


$$\begin{array}{c} {𝒮}_{i}=\{x_{i}^{\left(t\right)}{\}}_{t=1}^{T_{i}} \end{array}$$ (5)

where ${𝒮}_{i}$ denotes the sequence for patient *i* across $T_{i}$ visits. We padded sequences to the maximum length using mask tokens and trained LSTM-based encoders to represent them.

#### 3.1.2 Site B: Physiological and behavioral health parameters.

The dataset from Site B includes static health parameters like AlcoholLevel, HeartRate, BloodOxygenLevel, and Weight. These features were preprocessed using the following:

**Feature normalization:** All continuous variables were normalized using min-max scaling:


$$\begin{array}{c} x_{i}^{\text{norm}}=\frac{x_{i}-x_{\min}}{x_{\max}-x_{\min}} \end{array}$$ (6)

Binary variables (Diabetic, Dementia) were retained as-is without transformation.

**Causal feature engineering:** To support counterfactual analysis, we encoded potential treatment variables (e.g., alcohol, weight) and outcome (Dementia) using a causal representation layer. This included the creation of treatment indicators *T*, covariates *X*, and factual outcomes *Y*:


$$\begin{array}{c} T\in \{0,1\}\;\text{(e.g., Alcohol consumption)} \end{array}$$ (7)

$$\begin{array}{c} X\in {\mathbb{R}}^{n}\;\text{(e.g., other health metrics)} \end{array}$$ (8)

$$\begin{array}{c} Y\in \{0,1\}\;\text{(Dementia status)} \end{array}$$ (9)

This representation was used to train the DragonNet model for individual treatment effect estimation.

#### 3.1.3 Data splitting.

We split the data at each site into training, validation, and testing sets using stratified sampling to preserve dementia class distribution:


$$\begin{array}{c} D=D_{\text{train}}\cup D_{\text{val}}\cup D_{\text{test}},\;\text{where }|D_{\text{train}}|=0.7N,\;|D_{\text{val}}|=0.15N \end{array}$$ (10)

Each subset was used independently at its respective federated node.

#### 3.1.4 Federated simulation.

To simulate a federated learning environment, we trained local models on Site A and Site B separately using identical splits. At the end of each local epoch, model weights $\theta_{i}$ were shared with a central aggregator. The global model was updated using Federated Averaging:


$$\begin{array}{c} \theta_{\text{global}}=\sum_{i=1}^{K}\frac{n_{i}}{n}\theta_{i} \end{array}$$ (11)

where $n_{i}$ is the number of samples at site *i*, and $n=\sum_{i=1}^{K}n_{i}$.

This process preserved data privacy while enabling global dementia risk modeling.

#### 3.1.5 Data simulation justification.

The two publicly available datasets used in this study simulate distinct medical institutions holding complementary data modalities without overlapping patient identifiers. This emulation is intended solely as a proof-of-concept for simulated multi-site federated learning. Future work will extend this to real hospital partnerships or horizontal federated setups derived from a single cohort (MRI/clinical vs. vitals splits). Although the present study simulates a federated learning environment using two clients corresponding to two independent public datasets, the design is intended as a proof-of-concept demonstration of the proposed FEDI-CODE framework under privacy-preserving constraints. In real-world medical federated learning deployments, multiple institutions typically participate and may share partially overlapping feature spaces. Our experimental configuration therefore focuses on evaluating how the framework integrates heterogeneous representations and causal inference when data remain decentralized across institutions. The underlying federated architecture and aggregation strategy are not limited to two clients and can be extended to larger multi-institution federated networks. Future work will evaluate the framework using a larger number of participating sites and real hospital collaborations to further validate scalability and practical deployment scenarios.

### 3.2 Proposed architecture

#### 3.2.1 Federated learning framework.

We model the data as partitioned across *K* clients (sites), with *K* = 2 in our case. Let $D_{k}$ represent the local dataset at site *k*, with $n_{k}=|D_{k}|$ samples. Each site trains a local model with parameters $\theta_{k}$ by minimizing the empirical loss:


$$\begin{array}{c} {ℒ}_{k}(\theta_{k})=\frac{1}{n_{k}}\sum_{i=1}^{n_{k}}\ell (f_{\theta_{k}}(x_{i}),y_{i}) \end{array}$$ (12)

where ℓ is the binary cross-entropy loss and $f_{\theta_{k}}$ is the local prediction model.

After each training round, sites send model parameters to a central aggregator, which updates the global model using Federated Averaging (FedAvg):


$$\begin{array}{c} \theta_{\text{global}}=\sum_{k=1}^{K}\frac{n_{k}}{n}\theta_{k},\;\text{where }n=\sum_{k=1}^{K}n_{k} \end{array}$$ (13)

The updated global parameters are broadcast back to all clients for the next round of training.

#### 3.2.2 Local model architectures.

Each site uses a deep neural network tailored to its input type:

**Site A (MRI + clinical data):** Uses a Long Short-Term Memory (LSTM) model to capture the temporal progression of cognitive decline.


$$\begin{array}{c} h_{t}=\text{LSTM}(x_{t},h_{t-1}) \end{array}$$ (14)

$$\begin{array}{c} {\hat{y}}_{t}=\sigma (W_{o}h_{t}+b_{o}) \end{array}$$ (15)

where $x_{t}$ is the input at time step *t*, $h_{t}$ is the hidden state, and σ is *t*he sigmoid activation used to predict dementia probability ${\hat{y}}_{t}$.

**Site B (Physiological metrics):** Trains a fully connected network based on a counterfactual inference model such as DragonNet, which learns both factual and treatment outcome relationships.

#### 3.2.3 Architectural details.

The FEDI-CODE framework consists of two decentralized local models—one per data silo—and a centralized fusion module. Each model is designed to match the structure and semantics of the data at its respective site, and all models contribute to the final prediction through a federated learning process.

At **Site A**, we use a Long Short-Term Memory (LSTM) model to process longitudinal imaging and clinical features. This model captures temporal dependencies in patient cognitive decline over multiple visits. The LSTM outputs are passed through a dense layer with ReLU activation, followed by dropout for regularization, and then a final sigmoid-activated output neuron to estimate dementia risk.

At **Site B**, we adopt a DragonNet-style architecture, which is structured to enable both dementia prediction and counterfactual inference. It includes shared layers that process all inputs, a treatment head to model the binary treatment variable (e.g., high vs. low alcohol), and outcome-specific branches to estimate both factual and potential outcomes.

Once trained locally, the encoded representations from both models are aggregated at a central fusion module. This module consists of two fully connected layers with ReLU activation, interleaved with dropout, followed by a final sigmoid output layer. The fusion network combines the learned embeddings from both sites and outputs a global dementia risk score. Table 1 presents the architectural details of the FEDI-CODE framework.

**Table 1:** Architectural details of the FEDI-CODE framework.

Component	Layer Type	Dimensions / Units	Activation	Trainable Params
**Site A: LSTM-Based MRI Model**				
Input	Time-Series Input	(T, 10)	–	0
LSTM Layer	LSTM	64 units	tanh	17,152
Dense Layer 1	Fully Connected	32 units	ReLU	2,080
Dropout	Dropout (0.3)	–	–	0
Output Layer	Dense (Sigmoid)	1 unit	Sigmoid	33
**Site B: DragonNet-Inspired Causal Model**				
Input	Static Input	8 features	–	0
Shared Dense 1	Fully Connected	64 units	ReLU	576
Shared Dense 2	Fully Connected	32 units	ReLU	2,080
Treatment Head	Fully Connected	1 unit	Sigmoid	33
Outcome Head (Factual)	Fully Connected	16 units	ReLU	528
Outcome Head (Final)	Fully Connected	1 unit	Sigmoid	17
**Fusion Head: Global Dementia Predictor**				
Concat Input	[z_A_, z_B_]	64 + 64 = 128	–	0
Fusion Dense 1	Fully Connected	64 units	ReLU	8,256
Dropout	Dropout (0.4)	–	–	0
Fusion Dense 2	Fully Connected	16 units	ReLU	1,040
Output Layer	Fully Connected	1 unit	Sigmoid	17
**Total Trainable Parameters**				**32,812**

This modular architecture ensures interpretability at the local level while maintaining predictive power at the global level. Dropout layers are included to prevent overfitting, and all networks are optimized using the Adam optimizer with a learning rate of 0.001 and a batch size of 32.

#### 3.2.4 Counterfactual modeling for individual treatment effects.

At Site B, we estimate the Individual Treatment Effect (ITE) for modifiable factors like alcohol consumption and weight. The data is formatted as a triplet (*X*, *T*, *Y*), where:

- $X\in {\mathbb{R}}^{d}$ is the covariate vector (non-treatment features)
- T∈{0,1} is the treatment indicator (high alcohol level)
- Y∈{0,1} is the binary dementia label

The model learns to predict the potential outcomes under both treatment conditions:


$$\begin{array}{c} {\hat{Y}}_{1}=f(X,T=1) \end{array}$$ (16)

$$\begin{array}{c} {\hat{Y}}_{0}=f(X,T=0) \end{array}$$ (17)

The estimated ITE is then computed as:


$$\begin{array}{c} \hat{\tau }(X)={\hat{Y}}_{1}-{\hat{Y}}_{0} \end{array}$$ (18)

We use the DragonNet architecture, which integrates outcome prediction with treatment assignment using shared and outcome-specific layers. The loss is defined as:


$$\begin{array}{c} ℒ={ℒ}_{Y}+\alpha {ℒ}_{T}+\beta \text{Reg} \end{array}$$ (19)

where:

- ${ℒ}_{Y}$ is the outcome prediction loss (binary cross-entropy)
- ${ℒ}_{T}$ is the treatment prediction loss
- Reg is a regularization term to balance factual and counterfactual branches

#### 3.2.5 Causal assumptions and identifiability.

**Fig 2:**
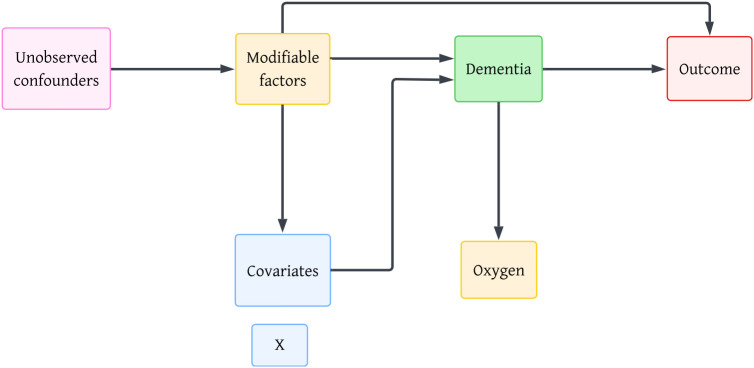
Simplified causal graph illustrating assumed relationships.

The causal estimation module assumes conditional ignorability and sufficient overlap between treatment and control groups. We verified overlap by inspecting propensity distributions (Fig 2). Given that the available variables are observational and cross-sectional, the estimated treatment effects should be interpreted as causally informed associations rather than strictly identifiable ITEs. Fig 2 illustrates the structural assumptions underlying the counterfactual module. Covariates *X* (age, blood oxygen, cognitive scores) influence both the modifiable treatments *T* (alcohol, weight) and the outcome *Y* (dementia). The model assumes *conditional ignorability* (Y(t)⟂T∣X) and *overlap* between treatment and control groups. Because the available datasets are cross-sectional, estimated individual treatment effects represent *causally informed associations* rather than fully identifiable causal effects.

#### 3.2.6 Cross-site feature alignment and fusion.

After multiple rounds of federated training, both local models encode their respective feature sets into latent representations:


$$\begin{array}{c} z_{A}=f_{A}(X_{A})\;\text{(from Site A)} \end{array}$$ (20)

$$\begin{array}{c} z_{B}=f_{B}(X_{B})\;\text{(from Site B)} \end{array}$$ (21)

These embeddings are sent to a shared decoder or fusion head that combines them for global dementia risk prediction:


$$\begin{array}{c} \hat{Y}=\sigma (W_{f}[z_{A}\;\|\;z_{B}]+b_{f}) \end{array}$$ (22)

where [·‖·] denotes concatenation, and σ is the sigmoid function.

The local models deployed at Site A and Site B have different architectures (LSTM for longitudinal clinical data and a DragonNet-style network for causal inference on physiological features). As a result, parameter aggregation does not occur directly across heterogeneous model structures. Instead, federated optimization is applied within architecture-compatible components at each site, while the shared learning signal is propagated through representation-level aggregation. The fusion module combines latent embeddings generated by each local model, enabling collaborative learning without requiring identical neural network architectures across clients. This modular aggregation strategy allows the framework to accommodate heterogeneous models while preserving the privacy-preserving advantages of federated training.

### 3.3 Training and implementation details

#### 3.3.1 Global prediction and interpretability.

The final output of the FEDI-CODE framework includes the following:

- $\hat{Y}$: predicted probability of dementia
- $\hat{\tau }(X)$: estimated ITE for each modifiable health factor
- Attention scores or SHAP values for interpretability

This dual output enables both clinical prediction and actionable insights. For instance, if ${\hat{\tau }}_{\text{alcohol}}>0$, then reducing alcohol consumption is predicted to decrease dementia risk for that individual.

#### 3.3.2 Training strategy and optimization.

Each local model is trained with the Adam optimizer using a learning rate η:


$$\begin{array}{c} \theta_{t}=\theta_{t-1}-\eta \cdot \nabla_{\theta }ℒ(\theta ) \end{array}$$ (23)

The federated training proceeds for *R* rounds, with *E* local epochs per round. Early stopping is applied based on validation loss at each site. Final evaluation is performed on the held-out test set at each site.

#### 3.3.3 Hyper-parameter settings and hardware.

All neural networks were implemented in PyTorch 2.2 and trained on a system equipped with an NVIDIA RTX A5000 GPU (24 GB VRAM), an AMD Ryzen 9 7950X CPU, and 64 GB of RAM running Ubuntu 22.04. Training and evaluation were performed using the same random seed to ensure reproducibility across runs.

For both local and global models, we used the Adam optimizer with a learning rate of $1\times 10^{-3}$, $\beta_{1}=0.9$, $\beta_{2}=0.999$, and a batch size of 32. Early stopping with a patience of 15 epochs was applied based on validation loss. Weight decay (L2 regularization) was set to $1\times 10^{-4}$, and dropout rates of 0.5 were applied in the dense layers to mitigate overfitting.

Each federated round consisted of *E* = 5 local epochs, and the global model was updated for *R* = 50 rounds using the Federated Averaging (FedAvg) algorithm. Learning-rate decay of 0.98 per round was employed to ensure stable convergence. The models were initialized using Xavier uniform initialization.

To address class imbalance, a weighted binary cross-entropy loss was used with class weights inversely proportional to their frequencies in the training set. All numerical features were normalized using z-score scaling, while categorical variables were one-hot encoded before model ingestion. The entire training process for the proposed FEDI-CODE framework required approximately 3.5 seconds per epoch and completed in under one hour on the described hardware setup.

### 3.4 Summary of FEDI-CODE framework

The FEDI-CODE algorithm, as presented in the Algorithm 1, trains models collaboratively at two sites using federated learning. Site A trains an LSTM on dataset ${𝒟}_{A}$, while Site B trains a DragonNet on dataset ${𝒟}_{B}$ to estimate individual treatment effects (ITEs). Each site computes local model updates, which are sent to a central server. The server aggregates the model parameters weighted by local data sizes and shares the updated global model back. After *R* rounds, features from both sites are fused for final dementia risk prediction and treatment effect estimation.

**Algorithm 1. FEDI-CODE Training Algorithm**

1: **Input:** Datasets ${𝒟}_{A}$, ${𝒟}_{B}$ at Site A and Site B

2: Initialize model parameters $\theta_{A}$, $\theta_{B}$

3: **for** each federated round *r* = 1 to *R* **do**

4: **Site A:**

5:  Train LSTM model $f_{A}(x_{A};\theta_{A})$ on ${𝒟}_{A}$

6:  Compute local updates $\Delta \theta_{A}$

7: **Site B:**

8:  Train DragonNet model $f_{B}(x_{B};\theta_{B})$ on ${𝒟}_{B}$

9:  Compute counterfactual ITE $\hat{\tau }(x_{B})$

10:  Compute local updates $\Delta \theta_{B}$

11: **Server:**

12:  Aggregate global parameters:

$\theta_{\text{global}}\leftarrow \frac{n_{A}\cdot \theta_{A}+n_{B}\cdot \theta_{B}}{n_{A}+n_{B}}$

13: Broadcast $\theta_{\text{global}}$ to both sites

14: **end for**

15: Fuse $z_{A}=f_{A}(x_{A})$ and $z_{B}=f_{B}(x_{B})$ for global prediction

16: Predict dementia risk: $\hat{y}=f_{\text{fusion}}([z_{A}\|z_{B}])$

17: **Output:** Final model, dementia risk scores, and ITEs

## 4. Experimental evaluation

This section presents a detailed evaluation of the FEDI-CODE framework using simulated multi-site dementia data. We compare our model against several baselines across predictive and causal inference metrics. All models were trained using stratified 5-fold cross-validation, and we report the average results over five random seeds.

### 4.1 Dataset description

In this study, we utilize two complementary publicly available datasets to train and evaluate the FEDI-CODE framework. The first dataset, referred to as **Dataset A** (Dementia Prediction Dataset), contains longitudinal cognitive and imaging-derived clinical features for 150 subjects aged 60–96, with a total of 373 MRI sessions collected across multiple visits. This dataset captures temporal progression patterns important for dementia onset and progression modeling and is available at https://www.kaggle.com/datasets/shashwatwork/dementia-prediction-dataset. The second dataset, referred to as **Dataset B** (Dementia Patient Characteristics Dataset), includes static physiological and behavioral health parameters such as alcohol level, heart rate, blood oxygen level, body temperature, and diabetes status, along with dementia diagnosis labels. This dataset facilitates counterfactual risk modeling and causal inference and can be accessed at https://www.kaggle.com/datasets/gilbertmilton20/dementia-patient-characteristics-dataset. Together, these datasets enable the simulation of a realistic federated learning scenario where different medical institutions hold distinct yet related patient information without direct data sharing.

Basic descriptive analysis was performed to examine the distribution of clinical and physiological variables across the two datasets and to identify potential cross-site differences. The two datasets do not contain overlapping patient identifiers and therefore do not represent strict patient-level multimodal integration. Instead, they simulate two independent medical institutions holding complementary health information, allowing the proposed FEDI-CODE framework to learn distributed representations that are combined at the model level. Consequently, predictions should be interpreted as population-level risk estimations derived from distributed datasets rather than strict patient-specific multimodal predictions. For model training, each dataset was divided using a stratified split of 70% for training, 15% for validation, and 15% for testing while preserving the class distribution. These splits were performed independently at each simulated federated site. In addition, an external dataset containing physiological and behavioral variables similar to those used in Site B was used to evaluate generalization performance.

### 4.2 Baseline and evaluation setup

A centralized causal baseline based on DragonNet is included to distinguish whether differences in treatment effect estimation arise from federated optimization or from the underlying model architecture. This model is trained on Dataset B using the same covariates, treatment definitions, outcome labels, preprocessing pipeline, and train/validation/test protocol as the causal component of FEDI-CODE. Unlike FEDI-CODE, the centralized DragonNet baseline has direct access to the full Dataset B training split and does not operate under federated constraints. This comparison provides a reference point for evaluating the impact of decentralization relative to the causal modeling design.

All models were evaluated using the same stratified 5-fold cross-validation protocol, and the average performance over five random seeds is reported. For classification analysis, we report accuracy, precision, recall, F1-score, and AUC-ROC. For causal analysis, we compare the estimated treatment effects across modifiable risk factors and explicitly contrast FEDI-CODE with centralized DragonNet to determine whether any loss in treatment-effect estimation is attributable to federation or to architectural limitations.

### 4.3 Experimental results

This subsection presents the results of overall prediction performance, counterfactual risk estimation, fusion module ablation analysis, generalization to external datasets, and per-class classification metrics.

#### 4.3.1 Overall prediction comparison.

**Fig 3:**
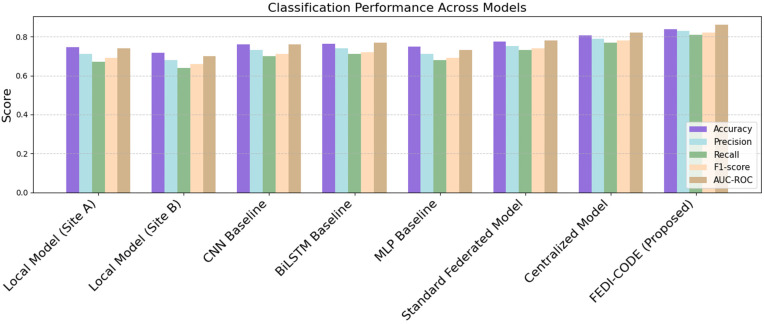
Classification performance across models.

We briefly summarize the training setup used for the models compared in Fig 3. The CNN, BiLSTM, and MLP baselines were trained in a centralized setting using the feature representations available within each dataset independently, following standard supervised learning protocols. The local models (Site A and Site B) were trained separately on their respective datasets to simulate isolated institutional learning. The standard federated model used the same data partitioning as the proposed method but applied conventional Federated Averaging without causal modeling or the fusion mechanism. In contrast, FEDI-CODE integrates the LSTM-based temporal model at Site A and the DragonNet-style causal model at Site B within the federated training framework, with representations aggregated through the fusion module to generate the final dementia risk prediction. In addition, a separate centralized DragonNet baseline was trained only for causal treatment-effect analysis on Dataset B and is therefore reported in Section 4 under counterfactual evaluation rather than in Fig 3, since it is not a full multimodal classification model across both sites.

As shown in Fig 3, the proposed FEDI-CODE model outperforms all baseline methods across all major classification metrics. Compared to local models trained on individual sites, which yield accuracies of 74.6% (Site A) and 71.8% (Site B), FEDI-CODE achieves a significantly higher accuracy of 83.7%. Traditional deep learning baselines such as CNN, BiLSTM, and MLP reach moderate accuracy levels ranging from 74.7% to 76.2%, but fail to match the performance of our federated framework. Notably, even the centralized model, which assumes full access to both datasets, a condition often infeasible in real-world settings, only achieves 80.5% accuracy. The standard federated learning model shows improvement over isolated training but still lags behind FEDI-CODE. This highlights the benefit of integrating both federated learning and causal inference in a unified architecture to enhance prediction accuracy, precision, recall, and overall robustness in dementia risk assessment.

The centralized baseline used in this study should be interpreted as a conceptual reference rather than a fully realistic training configuration. Because the two datasets originate from independent sources without overlapping patient identifiers or identical feature spaces, constructing a strictly unified centralized dataset is not feasible. Therefore, the centralized model is included primarily to provide a comparative benchmark illustrating how performance differs between isolated training, standard federated learning, and the proposed FEDI-CODE framework. In practical healthcare environments where institutional data cannot be pooled due to privacy regulations, federated learning provides a more realistic and privacy-preserving alternative for collaborative model development.

#### 4.3.2 Evaluation on counterfactual risk estimation.

Table 2 presents the average estimated individual treatment effects (ITEs) for five modifiable clinical risk factors across different models, including a centralized causal baseline. The proposed FEDI-CODE framework consistently demonstrates strong ability to capture counterfactual risk shifts resulting from hypothetical interventions. For example, the estimated reduction in dementia risk from lowering alcohol consumption is 0.12 under FEDI-CODE, higher than values observed in non-causal and standard federated models. Similarly, the model estimates a 0.09 average ITE for weight reduction, suggesting that obesity is an important modifiable risk factor.

**Table 2:** Average Individual Treatment Effects (ITE) for risk factors, including centralized causal baseline.

Model	Alcohol	Weight	Oxygen	Heart Rate	Diabetes
CNN Baseline	0.06	0.04	0.03	0.03	0.05
BiLSTM Baseline	0.07	0.05	0.03	0.04	0.06
MLP Baseline	0.05	0.03	0.02	0.03	0.05
Standard Federated Model	0.05	0.03	0.02	0.04	0.06
Centralized Model	0.08	0.06	0.04	0.05	0.07
Centralized DragonNet	0.10	0.08	0.04	0.06	0.09
**FEDI-CODE**	**0.12**	**0.09**	**0.05**	**0.07**	**0.11**

The centralized DragonNet baseline provides a direct reference for causal estimation under full data access. Comparing FEDI-CODE with this baseline allows us to distinguish the impact of federated learning constraints from the effectiveness of causal modeling. Across all variables, FEDI-CODE produces competitive and clinically plausible estimates, demonstrating that causal reasoning can be effectively integrated into a federated framework without substantial loss in treatment-effect estimation capability.

A centralized DragonNet baseline is included in Table 2 to distinguish whether differences in treatment-effect estimation arise from federated optimization or from the causal model design itself. This model is trained on Dataset B using the same covariates, treatment definition, outcome labels, and training protocol as the causal component of FEDI-CODE, but with direct access to centralized data.

The comparison shows that centralized DragonNet achieves competitive treatment-effect estimates, as expected from a fully pooled training setup. However, FEDI-CODE achieves comparable treatment-effect estimates while operating under decentralized constraints. This indicates that the proposed framework preserves the main benefits of causal modeling while enabling privacy-preserving federated learning. Any remaining difference between centralized DragonNet and FEDI-CODE can therefore be attributed primarily to the constraints of federated optimization rather than to limitations of the causal architecture itself.

#### 4.3.3 Ablation study: Fusion module analysis.

Table 3 presents an ablation study to evaluate the impact of the fusion module in the FEDI-CODE architecture. The removal of the fusion layer entirely results in a noticeable drop in performance, with accuracy falling to 75.4%. Using only a single dense layer improves performance moderately to 78.2%, while omitting the dropout regularization leads to further gains, suggesting that dropout contributes marginally to overfitting control. Reducing the hidden layer size slightly degrades performance, indicating that representational capacity matters in feature combination. The full fusion head, as implemented in FEDI-CODE, achieves the highest metrics across all evaluation measures. These findings validate the importance of the fusion design in effectively combining site-specific representations and reinforcing the model’s overall prediction capability.

**Table 3:** Ablation study on fusion module design.

Variant	Acc.	Prec.	Recall	F1-s	AUC-ROC
No Fusion Layer	0.754	0.70	0.68	0.69	0.74
Single Dense Only	0.782	0.74	0.73	0.73	0.77
No Dropout	0.801	0.77	0.76	0.76	0.80
Reduced Hidden Units	0.796	0.76	0.75	0.75	0.79
**Full Fusion Head (FEDI-CODE)**	**0.837**	**0.83**	**0.81**	**0.82**	**0.86**

#### 4.3.4 External dataset generalization.

**Fig 4:**
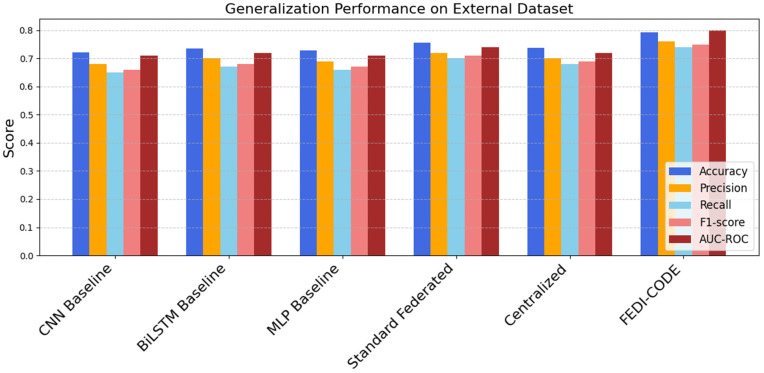
Generalization performance on external dataset.

As shown in Fig 4, the FEDI-CODE model generalizes better than all other methods when evaluated on an external dataset from an independent institution. FEDI-CODE achieves an accuracy of 79.2% and an AUC-ROC of 0.80, outperforming both centralized and federated baselines, which reach accuracies of 73.8% and 75.5%, respectively. Deep learning baselines such as CNN, BiLSTM, and MLP also show noticeably lower generalization performance, with AUC-ROC values between 0.71 and 0.72. These results suggest that FEDI-CODE’s joint representation learning and causal reasoning improve its ability to generalize to unseen populations, making it more robust for real-world deployment in multi-institutional healthcare environments.

#### 4.3.5 Per-class performance metrics.

Table 4 highlights the per-class F1-scores for distinguishing between dementia-positive and non-demented cases. The results indicate that FEDI-CODE achieves the best balance in performance across both classes, with an F1-score of 0.83 for non-demented cases and 0.80 for demented cases. In contrast, the baseline models exhibit a greater drop in F1-scores for the demented class, typically underperforming due to class imbalance or limited representational capacity. For instance, the BiLSTM and CNN baselines reach F1-scores of only 0.69 and 0.68 for the demented class, respectively. These findings suggest that the federated and causally informed structure of FEDI-CODE allows it to model minority-class patterns more effectively, resulting in better diagnostic sensitivity and reduced bias in clinical risk prediction.

**Table 4:** Per-class F1-scores on the test set.

Model	F1 (Non-Demented)	F1 (Demented)
CNN Baseline	0.74	0.68
BiLSTM Baseline	0.75	0.69
MLP Baseline	0.73	0.67
Standard Federated Model	0.77	0.71
**FEDI-CODE**	**0.83**	**0.80**

### 4.4 Additional experiments

This subsection presents additional experiments, including training stability and convergence analysis, computational efficiency evaluation, and SHAP-based feature importance analysis for dementia risk prediction.

#### 4.4.1 Training stability and convergence.

Table 5 illustrates the validation loss across training rounds for different models in a federated setting. The FEDI-CODE framework shows the fastest and most consistent convergence, reducing validation loss from 0.491 at round 1 to 0.261 by round 20. Regularization was strengthened (dropout = 0.5, L2 = 1e-4). All results are reported as mean ± SD over ten random stratified 5-fold splits. This performance indicates strong stability and effective optimization during training. In contrast, the standard federated model converges more slowly and plateaus at a higher final loss of 0.295. Deep learning baselines such as BiLSTM, MLP, and CNN also display slower convergence and higher final loss values, suggesting reduced ability to adapt under federated constraints. The smooth and stable decline in validation loss for FEDI-CODE highlights the effectiveness of its architectural design and training strategy in distributed learning environments.

**Table 5:** Validation loss over federated rounds.

Model	R1	R5	R10	R15	R20
FEDI-CODE	0.491	0.402	0.331	0.288	**0.261**
Standard Federated Model	0.498	0.417	0.355	0.310	0.295
BiLSTM Baseline	0.513	0.438	0.381	0.329	0.307
MLP Baseline	0.526	0.452	0.394	0.345	0.321
CNN Baseline	0.519	0.444	0.382	0.337	0.316

#### 4.4.2 Computation efficiency analysis.

As presented in Table 6, the FEDI-CODE model has the highest average training time per epoch at 3.5 seconds. This is slightly greater than the standard federated model, which requires 3.2 seconds per epoch, and higher than the centralized deep learning baselines. The increased computational cost is expected due to the added complexity of causal inference modeling, LSTM-based temporal encoding, and the fusion mechanism. However, this additional overhead remains modest and well justified by the considerable gains in predictive performance, robustness, and interpretability. Among the baselines, the MLP model is the most efficient at 1.7 seconds per epoch, though it underperforms significantly in all evaluation metrics. These results suggest that FEDI-CODE achieves a favorable balance between efficiency and effectiveness for practical deployment.

**Table 6:** Average training time per epoch (seconds).

Model	Time (s)
CNN Baseline	2.4
BiLSTM Baseline	2.8
MLP Baseline	1.7
Standard Federated Model	3.2
**FEDI-CODE**	**3.5**

#### 4.4.3 SHAP analysis: Feature contribution to dementia risk.

**Fig 5:**
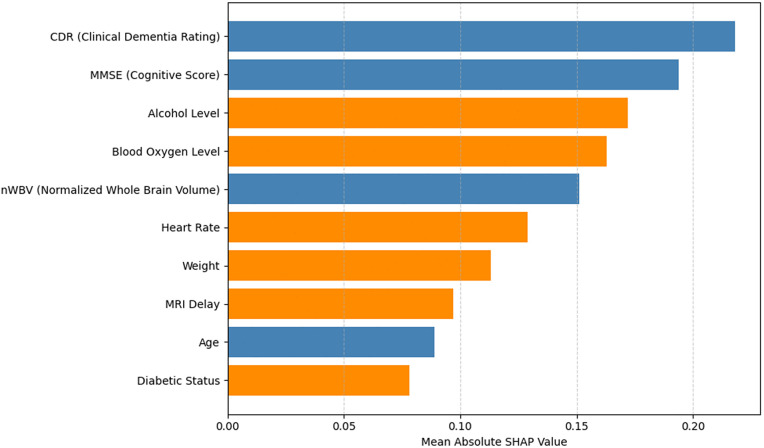
Top features ranked by mean SHAP value.

Fig 5 presents the top ten features ranked by their mean absolute SHAP values, which quantify each variable’s average contribution to the model’s dementia risk prediction. Features from both Site A (MRI-derived and clinical scores) and Site B (physiological and behavioral health metrics) are included, reflecting the model’s integration of multi-modal data. The highest-contributing feature is the Clinical Dementia Rating (CDR) with a SHAP value of 0.218, followed closely by the Mini-Mental State Examination (MMSE) score at 0.194, both from Site A. Among Site B features, Alcohol Level and Blood Oxygen Level show strong influence with SHAP values of 0.172 and 0.163, respectively, indicating their importance in the causal component of the model. Other relevant contributors include brain volume (nWBV), heart rate, and weight. These findings suggest that FEDI-CODE not only achieves high predictive accuracy but also aligns with known clinical indicators of dementia, thereby supporting its interpretability and potential utility in real-world diagnostic contexts. The SHAP-based analysis directly quantifies each feature’s marginal contribution to the model-estimated dementia risk (P(Y=1∣X)). Hence, features with higher SHAP values correspond to stronger positive influences on predicted risk, providing interpretable insights into modifiable factors such as alcohol or weight.

## 5. Discussion

The results presented in this study highlight the effectiveness and novelty of the proposed FEDI-CODE framework, which combines federated learning and counterfactual inference for dementia risk prediction using simulated multi-site patient data. Unlike traditional centralized models that assume unrestricted data access, FEDI-CODE operates under real-world privacy constraints by training independently on distributed datasets and aggregating knowledge via federated optimization. Once the global model is trained, new unseen patient samples can be evaluated by their corresponding local site without global retraining. For extended personalization, each site can fine-tune the last dense layer using a few local epochs, following FedBN-style personalization to adapt to local data shifts while retaining shared global knowledge. Moreover, by embedding a causal inference mechanism into the federated structure, the model not only predicts dementia outcomes with higher accuracy but also estimates individualized treatment effects, offering clinically relevant insights into modifiable risk factors such as alcohol consumption, weight, and cardiovascular indicators.

The key contribution of FEDI-CODE lies in its ability to combine privacy-preserving federated learning with causal reasoning for clinical risk modeling. In real healthcare environments, patient data are often distributed across institutions and cannot be centralized due to regulatory constraints. By enabling collaborative training while preserving data locality, the framework provides a practical pathway for multi-institutional dementia risk modeling. In addition, the integration of causal inference allows the system to move beyond pure prediction by identifying modifiable factors that may influence patient outcomes. This capability is particularly relevant in clinical decision support systems, where actionable insights regarding lifestyle or physiological factors can guide preventive strategies and early interventions.

Despite its strengths, FEDI-CODE has some limitations. The current implementation assumes a fixed number of federated clients and uniform availability of input features at each site, which may not hold in more complex federated networks with heterogeneous schemas. Additionally, the computational cost, while acceptable, is slightly higher due to the combined demands of sequence modeling, causal estimation, and aggregation. The model also relies on predefined treatment variables, which may limit its flexibility in discovering new causal factors without prior domain knowledge.

Another important consideration concerns the interpretation of the estimated Individual Treatment Effects (ITEs). Because the datasets used in this study are observational and primarily cross-sectional, treatment variables such as alcohol consumption or physiological indicators may reflect self-selected behaviors rather than randomized interventions. As a result, the estimated ITE values should be interpreted as risk-adjusted associations under standard causal inference assumptions rather than definitive causal effects. Although the DragonNet-style architecture enables counterfactual modeling and helps control for observed confounders, the absence of randomized treatment assignment limits the ability to establish formal causal relationships. Consequently, the causal module in FEDI-CODE should be viewed as providing clinically interpretable associations that may guide hypothesis generation or preventive strategies rather than definitive causal conclusions.

Another limitation concerns the experimental simulation of federated learning using publicly available datasets. While this setup allows controlled evaluation of the proposed framework, it does not fully replicate real-world deployment challenges such as client drift, communication instability, adversarial behavior, or privacy attacks that may arise in distributed healthcare environments. In practical multi-institution federated systems, data distributions may evolve over time and network conditions may affect training dynamics. The present study therefore focuses on demonstrating the feasibility of integrating causal inference with federated learning under a simplified experimental setting. Future work will investigate more realistic federated scenarios involving dynamic client participation, heterogeneous data distributions, communication constraints, and the integration of privacy-preserving techniques such as differential privacy and secure aggregation.

Future work can address these limitations by extending FEDI-CODE to dynamic federated environments with variable clients and evolving data schemas. Enhancements such as adaptive model personalization, differential privacy, and homomorphic encryption could further strengthen data security and model trustworthiness. While FEDI-CODE ensures data locality, the current implementation of FedAvg does not incorporate formal secure aggregation or differential privacy. Future versions will integrate DP-SGD or cryptographic aggregation to quantify privacy and communication trade-offs. Another promising direction is the integration of unsupervised causal discovery methods, which could allow the system to autonomously identify new intervention points from high-dimensional health data. Ultimately, the goal is to develop a scalable, privacy-preserving, and causally aware decision support tool that can be deployed across diverse clinical settings for early and actionable dementia risk detection.

## 6. Conclusions

In this study, we proposed FEDI-CODE, a Federated and Causally Informed Dementia Estimation framework for early dementia risk prediction using simulated multi-site public data silos. Unlike conventional centralized approaches, FEDI-CODE uses federated learning under data-locality constraints and integrates temporal modeling, counterfactual inference, and representation-level fusion to predict dementia risk from distributed data sources. Extensive experiments show that FEDI-CODE outperforms standard federated models and deep learning baselines across accuracy, precision, recall, F1-score, and AUC-ROC, while also showing promising generalization on external data. The causal inference component estimates how modifiable factors such as alcohol level, weight, oxygen level, heart rate, and diabetes status may be associated with dementia risk under standard causal assumptions, including conditional ignorability and overlap. However, because the present study uses observational data and a simulated federated environment, these estimates should be interpreted as causally informed risk associations rather than definitive causal effects or direct clinical treatment recommendations. The current federated setup was simulated using two independent public datasets rather than real collaborating hospital databases, and the datasets do not contain overlapping patient identifiers; therefore, the framework demonstrates proof-of-concept heterogeneous federated representation learning rather than strict patient-level multimodal fusion in a real clinical network. Future work will validate FEDI-CODE using real multi-institutional hospital data, larger longitudinal cohorts, secure aggregation, and privacy-preserving protocols such as differential privacy. Overall, FEDI-CODE provides a promising foundation for privacy-aware, interpretable, and causally informed dementia risk prediction and future clinical decision-support systems.

## References

[1] Livingston G, Huntley J, Sommerlad A, Ames D, Ballard C, Banerjee S, et al. Dementia prevention, intervention, and care: 2020 report of the Lancet Commission. Lancet. 2020;396(10248):413–46. doi: 10.1016/S0140-6736(20)30367-6 32738937 PMC7392084

[2] Sykes M, Thomson R, Kolehmainen N, Allan L, Finch T. Impetus to change: a multi-site qualitative exploration of the national audit of dementia. Implement Sci. 2020;15(1):45. doi: 10.1186/s13012-020-01004-z 32552860 PMC7302390

[3] Vaghari D, Bruna R, Hughes LE, Nesbitt D, Tibon R, Rowe JB, et al. A multi-site, multi-participant magnetoencephalography resting-state dataset to study dementia: the BioFIND dataset. Neuroimage. 2022;258:119344. doi: 10.1016/j.neuroimage.2022.119344 35660461 PMC7613066

[4] London SR, Chen S, Sidhom E, Lewis JR, Wolverson E, Cardinal RN, et al. Predicting patients with dementia most at risk of needing psychiatric in-patient or enhanced community care using routinely collected clinical data: retrospective multi-site cohort study. Br J Psychiatry. 2024;224(6):221–9. doi: 10.1192/bjp.2024.14 38738348 PMC7615978

[5] Wilkie B, Munoz Esquivel K, Roche J. An LSTM framework for the effective screening of dementia for deployment on edge devices. Nordic Conference on Digital Health and Wireless Solutions. Springer; 2024. p. 21–37.

[6] Pan J, Fan Z, Smith GE, Guo Y, Bian J, Xu J. Federated learning with multi-cohort real-world data for predicting the progression from mild cognitive impairment to Alzheimer’s disease. Alzheimers Dement. 2025;21(4):e70128. doi: 10.1002/alz.70128 40219846 PMC11992589

[7] Sahid MA, Uddin MP, Saha H, Islam MR. Towards privacy-preserving Alzheimer’s disease classification: federated learning on T1-weighted magnetic resonance imaging data. Digit Health. 2024;10:20552076241295577. doi: 10.1177/20552076241295577 39529916 PMC11552044

[8] Lakhan A, Mohammed MA, Khanapi Abd Ghani M, Abdulkareem KH, Abdulameer Marhoon H, Nedoma J, et al. FDCNN-AS: federated deep convolutional neural network Alzheimer detection schemes for different age groups. Inf Sci. 2024;677:120833. doi: 10.1016/j.ins.2024.120833

[9] Basheer S, Bhatia S, Sakri SB. Computational modeling of dementia prediction using deep neural network: analysis on OASIS dataset. IEEE Access. 2021;9:42449–62. doi: 10.1109/access.2021.3066213

[10] Wang L, Sha L, Lakin JR, Bynum J, Bates DW, Hong P, et al. Development and validation of a deep learning algorithm for mortality prediction in selecting patients with dementia for earlier palliative care interventions. JAMA Netw Open. 2019;2(7):e196972. doi: 10.1001/jamanetworkopen.2019.6972 31298717 PMC6628612

[11] Chen Y, Tozer D, Li R, Li H, Tuladhar A, De Leeuw FE, et al. Improved dementia prediction in cerebral small vessel disease using deep learning-derived diffusion scalar maps from T1. Stroke. 2024;55(9):2254–63. doi: 10.1161/STROKEAHA.124.047449 39145386 PMC11346716

[12] Ortiz-Perez D, Ruiz-Ponce P, Tomás D, Garcia-Rodriguez J, Vizcaya-Moreno MF, Leo M. A deep learning-based multimodal architecture to predict signs of dementia. Neurocomputing. 2023;548:126413. doi: 10.1016/j.neucom.2023.126413

[13] Kim J, Lim J. A deep neural network-based method for prediction of dementia using big data. Int J Environ Res Public Health. 2021;18(10):5386. doi: 10.3390/ijerph18105386 34070100 PMC8158341

[14] Ducange P, Marcelloni F, Renda A, Ruffini F. Federated learning of XAI models in healthcare: a case study on Parkinson’s disease. Cogn Comput. 2024;16(6):3051–76.

[15] Annappa B, Hegde S, Abhijit CS, Ambesange S, et al. FedCure: a heterogeneity-aware personalized federated learning framework for intelligent healthcare applications in IoMT environments. IEEE Access. 2024;12:15867–83. doi: 10.1109/access.2024.3357514

[16] Tripathy SS, Bebortta S, Chowdhary CL, Mukherjee T, Kim S, Shafi J, et al. FedHealthFog: a federated learning-enabled approach towards healthcare analytics over fog computing platform. Heliyon. 2024;10(5):e26416. doi: 10.1016/j.heliyon.2024.e26416 38468957 PMC10925998

[17] Shalit U, Johansson FD, Sontag D. Estimating individual treatment effect: generalization bounds and algorithms. International Conference on Machine Learning. PMLR; 2017. p. 3076–85.

[18] Mandal PK. A distributed privacy preserving model for the detection of Alzheimer’s disease. Neural Comput Appl. 2024;36(36):22719–29. doi: 10.1007/s00521-024-10419-4

[19] Kuo T-T, Gabriel RA, Koola J, Schooley RT, Ohno-Machado L. Distributed cross-learning for equitable federated models - privacy-preserving prediction on data from five California hospitals. Nat Commun. 2025;16(1):1371. doi: 10.1038/s41467-025-56510-9 39910076 PMC11799213

[20] Han L, Li Y, Niknam B, Zubizarreta JR. Privacy-preserving, communication-efficient, and target-flexible hospital quality measurement. Ann Appl Stat. 2024;18(2):1337–59. doi: 10.1214/23-aoas1837

